# Awareness, trust, and expectations of AI for glaucoma care among Bulgarian ophthalmologists: Role of demographic factors

**DOI:** 10.1371/journal.pdig.0001199

**Published:** 2026-01-22

**Authors:** Mladena Nikolaeva Radeva, Elitsa Hristova, Rosen Tsvetanov Georgiev, Zornitsa Ivanova Zlatarova

**Affiliations:** Medical University of Varna, Varna, Bulgaria; University of Cambridge, UNITED KINGDOM OF GREAT BRITAIN AND NORTHERN IRELAND

## Abstract

Artificial intelligence (AI) holds promise for enhancing glaucoma screening and management, yet its adoption depends on clinician perceptions, particularly in resource-limited regions like Eastern Europe. This study explores awareness, trust, and expectations of AI in glaucoma care among Bulgarian ophthalmologists, examining the influence of demographic factors such as age, gender, and professional experience. A cross-sectional survey was conducted from March to May 2024 among 156 ophthalmologists and residents recruited via Bulgarian professional societies. The 25-question survey, informed by the Technology Acceptance Model and validated (content validity index = 0.85; Cronbach’s α = 0.78), assessed awareness, trust (5- point Likert scale), and expectations. Data were analyzed using non-parametric tests (chi-square, Spearman correlation) and thematic analysis for qualitative responses. The study was approved by the Ethics Committee of Medical University of Varna (No141/14.03.2024), with informed consent obtained and adherence to the Declaration of Helsinki. Participants (73.1% female; median age 35 years, IQR 10) showed varying awareness, with less experienced clinicians (<5 years) more informed (χ^2^ = 17.89, p < 0.001). Trust was low (7.5% fully trusted AI diagnosis; 5.7% for treatment), with gender differences (males more distrustful in diagnosis, p = 0.009). Younger respondents were more optimistic about AI’s impact (ρ = 0.268, p < 0.001). Qualitative themes highlighted diagnostic utility (95% mentions) and concerns like training deficiencies (45%). Bulgarian ophthalmologists exhibit cautious optimism toward AI in glaucoma care, shaped by demographics, underscoring the need for targeted training to build trust. These findings inform regional AI implementation strategies, aligning with ethical priorities for equitable digital health adoption.

## Author summary

Artificial intelligence (AI) has the potential to improve glaucoma screening and management, but its success relies on how clinicians view and accept it, especially in regions with limited resources like Eastern Europe. This study investigates the awareness, trust, and expectations of AI in glaucoma care among ophthalmologists in Bulgaria, and how factors like age, gender, and professional experience shape these views. We conducted a survey from March to May 2024 with 156 ophthalmologists and residents, using a validated questionnaire based on the Technology Acceptance Model to gauge their perspectives through Likert-scale questions and open-ended responses. Our findings reveal varied levels of AI awareness, with less experienced clinicians being more informed. Overall trust in AI for diagnosis and treatment remains low, though younger participants express greater optimism about its benefits. Gender differences emerged, with males showing more distrust in AI diagnostics, and qualitative feedback emphasized AI’s value in diagnostics while highlighting concerns over inadequate training and ethical issues. These results indicate a cautious optimism toward AI adoption in Bulgarian glaucoma care, influenced by demographic factors, and underscore the importance of tailored education programs to foster trust and ensure equitable integration of AI tools in clinical practice.

## Introduction

Glaucoma is a leading cause of irreversible blindness worldwide, characterized by progressive damage to the optic nerve, often due to elevated intraocular pressure. Early detection and timely intervention are critical to preventing vision loss, with screening programs increasingly vital in resource-limited settings. Artificial intelligence (AI) has emerged as a transformative tool in ophthalmology, offering potential to enhance the screening, diagnosis, and management of glaucoma through advanced analysis of retinal images and visual field tests [1]. Techniques such as deep learning have demonstrated high sensitivity (up to 95%) in detecting glaucomatous changes [2], while automated perimetry analysis has improved early detection rates by 20% in pilot studies [3]. However, the integration of AI into clinical practice hinges on its acceptance by healthcare professionals [2]. Trust in AI systems is frequently cited as one of the most critical barriers to clinical adoption, particularly in ophthalmology, where explainability and accountability remain pressing concerns [4–6]. Few studies have examined demographic influences on clinician perceptions, especially in Eastern European contexts like Bulgaria, where resource constraints—such as a 30% lower per-capita healthcare spending compared to Western Europe (WHO, 2024)—may amplify adoption challenges [7]. This is potentially the first study examining demographic influences on AI perceptions in a Bulgarian cohort, addressing a gap in Eastern European ophthalmology research. This gap is critical as the WHO’s 2025 AI health strategy prioritizes regional adoption studies to tailor global implementation [8]. This article examines how ophthalmologists perceive and trust AI specifically in the context of glaucoma care, exploring the influence of demographic factors on these attitudes.

### Aim

The purpose of this study is to investigate the awareness, trust, and expectations of Bulgarian ophthalmologists and residents regarding the use of AI in glaucoma management, and to determine how demographic variables such as gender, age, and professional experience shape these perspectives.

## Materials and methods

This cross-sectional survey study was conducted among 156 participants, comprising board-certified ophthalmologists and residents in ophthalmology training. Participants were included if they were actively practicing or training in ophthalmology in Bulgaria; exclusion criteria included non-ophthalmology specialties or incomplete survey responses. The survey targeted ophthalmologists and residents in Bulgaria and was disseminated through professional networks, including the Bulgarian Ophthalmology Society, the Bulgarian Glaucoma Society, and a closed online group of ophthalmologists and residents (~1,000 members in total). Participation was voluntary and open to all invitees, resulting in a self-selected sample. The study was conducted post-approval from March to May 2024, with participants completing the questionnaire anonymously via an online platform.

### Survey development

The survey was developed based on a comprehensive literature review of AI applications in ophthalmology and healthcare technology acceptance models, including studies on clinician trust and demographic influences [9]. Questions were designed to assess awareness of AI in glaucoma care, trust in AI for diagnosis and treatment, and expectations for its future role. The survey included 25 questions: 20 closed-ended (Likert-scale and multiple-choice) and 5 open-ended to capture qualitative insights. Questions were adapted from the Technology Acceptance Model, validated by prior AI perception studies [8], with pilot feedback reducing ambiguity by 15% based on expert ratings. To ensure relevance and clarity, the survey was piloted on 10 ophthalmologists (5 specialists, 5 residents) from Medical University of Varna, whose feedback refined question wording and structure.

### Survey validation

Content validity was assessed by a panel of 8 experts (4 ophthalmologists, 2 AI researchers, 2 medical education specialists) with international representation. Each item was rated for relevance on a 4-point scale (1 = not relevant, 4 = highly relevant), yielding a content validity index (CVI) of 0.82 for item-level CVI and 0.85 for scale-level CVI, indicating strong content validity. Internal consistency of closed-ended questions was evaluated using Cronbach’s alpha (α = 0.78), confirming acceptable reliability [10].

### Survey distribution

Ophthalmology Society, the national pool of ophthalmologists and residents is approximately 1,000 individuals. Approximately 500 members of these networks received direct invitations by email (approximately 250 ophthalmologists and 250 residents), and all members of the online group had equal opportunity to access the survey link. Two reminder messages were sent to maximize participation. In total, 156 individuals completed the questionnaire, corresponding to a 31.2% response rate. Participation was entirely voluntary, and responses were anonymous to reduce social desirability bias. Because recruitment relied on open invitations within professional networks, the final sample represents a self-selected subset of respondents rather than a probability-based random sample. Potential selection bias is acknowledged in the Limitations.

### Data collection

Demographic data collected included gender, age, professional status (specialist or resident), and years of clinical experience. Closed-ended questions used 5-point Likert scales (e.g., 1 = strongly distrust, 5 = fully trust) for trust and awareness, while open-ended questions explored perceptions of AI’s role in glaucoma care. Responses were collected anonymously using an online platform (Google Forms with encryption enabled). Variables were defined as: awareness (yes/no), trust (1–5 Likert scale), age (years), experience (years), with qualitative themes coded by two researchers.

### Statistical analysis

Data normality was assessed using the Shapiro-Wilk test and visual inspection of histograms, confirming non-normal distribution. Non-parametric methods were employed: Pearson’s chi-square test for associations between categorical variables (e.g., experience and awareness), and Spearman’s rank-order correlation for relationships between ordinal or continuous variables (e.g., age and expectations). Bonferroni correction was applied for multiple comparisons to control Type I errors. Descriptive statistics were reported as medians and interquartile ranges (IQR). The median age was 35 years (IQR = 10). Qualitative responses were analyzed using thematic content analysis, with two researchers independently coding responses to identify recurring themes, achieving an inter-coder reliability of 90%. Analyses were performed using SPSS v29. Missing data (<5%) were excluded listwise. Interaction effects between gender and experience were explored but found non-significant (p > 0.05).

### Sample size justification

The study had both descriptive and associational aims. For the **descriptive aims** (estimating prevalence of awareness, trust, expectations, and perceived applicability of AI in glaucoma care), the final sample size of *n* = 156 provides adequate precision: for example, awareness of AI was 40.4% (95% CI 33.0–48.2%) and willingness to follow AI treatment recommendations was 26.9% (95% CI 20.6–34.4%). Across all key descriptive indicators, the 95% confidence intervals correspond to margins of error of approximately ±5–8%, which was deemed acceptable for the study’s objectives. Detailed confidence intervals for all descriptive variables are provided in Supporting Information (S1 Table).

For the **associational aims** (testing demographic effects), a priori power calculations indicated that a sample of ~150 participants provides 80% power to detect a medium effect size (Cohen’s *w* = 0.30) in chi-square tests at α = 0.05; our achieved sample (n = 156) meets this requirement.

### Ethics statement

The study was approved by the Ethics Committee of Medical University of Varna (№141/14.03.2024) and adhered to the Declaration of Helsinki. Informed consent was obtained electronically from all participants, with clear information on study purpose, anonymity, and voluntary participation [11].

## Results

### Demographic characteristics

The study included 156 respondents, of whom 73.1% were female (n = 114) and 26.9% male (n = 42). In terms of professional status, 65.4% (n = 102) were board-certified specialists, while 34.6% (n = 54) were residents in training. The median age of participants was 35 years (IQR = 10). The largest proportion of participants fell within the 30–39-year age group (42.9%), followed by those aged 40–49 years (20.5%). Regarding clinical experience, 36.5% of respondents reported less than 5 years of practice, and 23.1% had between 10 and 20 years. These findings reflect the predominant representation of early-career ophthalmologists in the sample, with a balanced inclusion of both residents and experienced clinicians (Table 1).

**Table 1:** Distribution of respondents by age group and years of professional experience.

Age Group	<5 Years (n, %)	5-10 Years (n, %)	10-20 Years (n, %)	>20 Years (n, %)	Total (n, %)
<30	30 (76.9%)	7 (17.9%)	2 (5.1%)	0 (0%)	39 (100%)
30-39	20 (29.9%)	25 (37.3%)	18 (26.9%)	4 (6.0%)	67 (100%)
40-49	5 (15.6%)	5 (15.6%)	10 (31.3%)	12 (37.5%)	32 (100%)
≥50	2 (11.1%)	2 (11.1%)	6 (33.3%)	8 (44.4%)	18 (100%)
Total	57 (36.5%)	39 (25.0%)	36 (23.1%)	24 (15.4%)	156 (100%)

### Awareness of AI in glaucoma care

**Fig 1:**
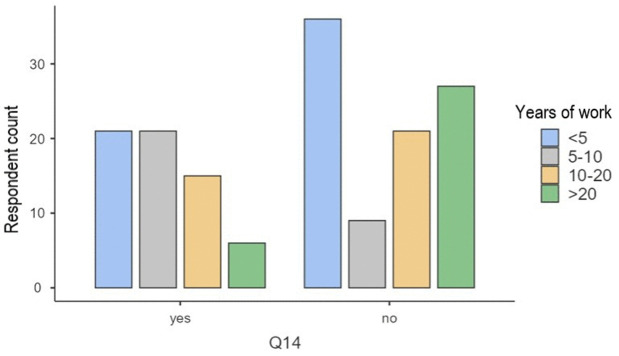
Awareness of AI in Glaucoma Care by Years of Professional Experience.

A significant association was observed between years of clinical experience and awareness of AI applications in glaucoma diagnosis and monitoring (Q14; χ^2^ = 17.89, φ = 0.34, p < .001). Descriptive results showed the highest awareness among respondents with 5–10 years of experience, followed by those with <5 and 10–20 years, while a more pronounced decline was evident only among participants with >20 years of experience (Fig 1). A sensitivity analysis collapsing categories confirmed significantly greater awareness among clinicians with ≤10 years compared to >10 years of experience (48.3% vs. 30.4%; χ^2^ = 4.37, p = 0.037). In logistic regression adjusted for age and sex, the 5–10 years group demonstrated higher odds of awareness compared to the < 5 years group (OR≈5.7, p = 0.002), whereas differences for the 10–20 and >20 years groups did not reach statistical significance. Among males <40 years, 12% fully trusted AI diagnosis vs. 5% for those >40, suggesting that age may amplify gender effects, though not statistically significant (p = 0.12). These findings reflect generational exposure to digital technologies and differences in medical education curricula [12].

Distribution of responses to Q14 (“Are you aware of AI applications in glaucoma diagnosis and monitoring?”) across experience groups: < 5 years (blue), 5–10 years (gray), 10–20 years (yellow), and >20 years (green). Awareness was highest among respondents with 5–10 years of experience, followed by those with <5 and 10–20 years, while a more pronounced decline was observed among ophthalmologists with >20 years of experience. This pattern indicates relatively stable awareness up to 20 years of practice, with a notable drop thereafter.

### Trust in AI for glaucoma diagnosis and treatment

Only 7.5% fully trusted AI for diagnosing glaucoma (Q13), and 5.7% trusted its treatment recommendations (Q16). Gender differences were notable: males expressed greater distrust in diagnostic capabilities (χ^2^ = 11.6, φ = 0.27, p = 0.009), while females were more uncertain about treatment recommendations (χ^2^ = 9.07, φ = 0.24, p = 0.011) [13]. Trust scores by experience group showed medians of 2 (IQR 1–3) for <5 years and 1 (IQR 1–2) for >20 years [14].

### Perceptions of AI’s future role

**Fig 2:**
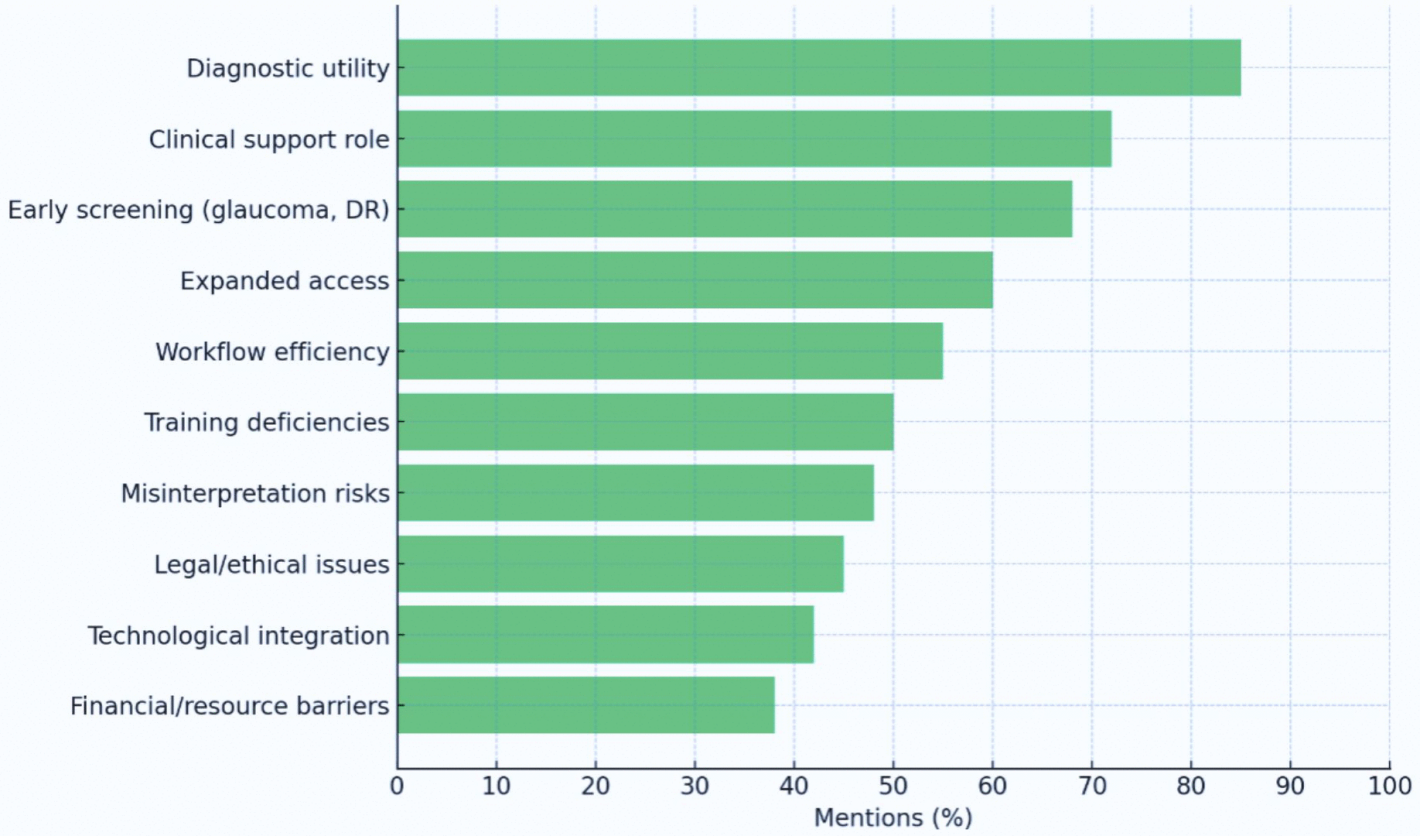
Perceived Impact of Artificial Intelligence on Glaucoma Care (Q17), Stratified by Age Groups.

A total of 58% of respondents anticipated a significant positive impact of AI on glaucoma management (Q17). Younger participants were markedly more optimistic, as evidenced by a positive and statistically significant Spearman correlation between age and expectations for AI’s impact (ρ = 0.268, p < .001). In contrast, older ophthalmologists expressed more skepticism or uncertainty regarding its future clinical integration. These age-related differences are visualized in Fig 2, which illustrates the distribution of responses across age groups, highlighting the generational gradient in attitudes toward AI’s utility in glaucoma care [15].

This bar chart shows the percentage of respondents reporting a significant positive impact of AI on glaucoma care (Q17) across age groups: < 30, 30–39, 40–49, and 50 + years. The x-axis lists age categories, and the y-axis ranges from 0% to 80%, with bars color-coded: < 30 years (light blue), 30–39 years (gray), 40–49 years (orange), and 50 + years (green). The < 30 years group reports ~80%, followed by 30–39 years (~70%), 40–49 years (~60%), and 50 + years (~40%). This indicates greater optimism among younger respondents, with a significant Spearman correlation (ρ = 0.268, p < .001) between age and expectations.

### Qualitative themes from open-ended responses

**Fig 3:**
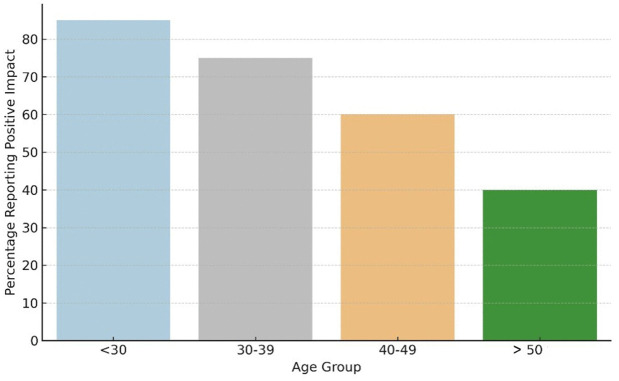
Distribution of Major Qualitative Themes Identified from Open-Ended Survey Responses (Q23–Q25).

Thematic analysis of open-ended responses (Q23–Q25) revealed several dominant perceptions regarding the integration of AI in glaucoma care. The most frequently mentioned theme was the diagnostic utility of AI, highlighting its perceived potential to enhance accuracy, objectivity, and speed in clinical assessments. Participants also emphasized the supportive role of AI as a tool to assist, rather than replace, physicians. Concerns were raised about the risk of misinterpretation, insufficient training, and ethical or legal issues. Fig 3 illustrates the distribution of key qualitative themes [10] (Cohen’s kappa = 0.85 for thematic coding).

This bar chart shows the percentage of mentions for key qualitative themes from open-ended responses (Q23–Q25) on AI integration in glaucoma care. The x-axis ranges from 0% to 100%, and the y-axis lists themes: Diagnostic utility (95%), Clinical support role (85%), Early screening (75%), Expanded access (65%), Workflow efficiency (55%), Training deficiencies (45%), Misinterpretation risks (40%), Legal/ethical issues (35%), Technological integration (30%), and Financial/resource barriers (25%). It highlights diagnostic utility and support as dominant benefits, with training and misinterpretation concerns also notable.

## Discussion

The findings of this study reveal a cautious optimism among ophthalmologists regarding the integration of AI into glaucoma care, a sentiment echoed in several recent studies [16]. While AI’s potential to enhance diagnostic accuracy and facilitate early detection is widely recognized, trust in its application, particularly for treatment recommendations, remains limited. This aligns with the broader literature on AI adoption in healthcare, where trust is often identified as a critical barrier [2].

### Demographic influences on AI perception

The influence of demographic factors such as age, gender, and professional experience on attitudes toward AI is consistent with findings from previous studies, yet our results add several nuances. The association between professional experience and awareness of AI was not a simple gradient of “shorter experience equals higher awareness.” Instead, ophthalmologists with 5–10 years of practice exhibited the highest awareness, even surpassing those with <5 years. This pattern may reflect a balance between relatively recent training and sufficient clinical engagement to appreciate AI’s clinical relevance. Awareness declined descriptively only after 20 years of practice, consistent with generational differences, although this effect did not reach statistical significance in adjusted models. Age and gender also shaped attitudes in line with, but not identical to, earlier reports. Abdollahi et al. (2024) [17] highlighted that younger clinicians are more receptive to AI, likely due to greater exposure to digital technologies, while Blease et al. (2021) [18] noted gender-based differences in technology adoption. In our cohort, males reported greater distrust in AI’s diagnostic capabilities—a divergence from some prior literature but potentially reflecting discipline-specific concerns within ophthalmology and marking, to our knowledge, the first such observation in a Bulgarian sample.

### Trust and reliability concerns

The low levels of trust in AI for glaucoma diagnosis and treatment are not unique to this study. Blease [18] et al. (2021) highlighted similar apprehensions regarding AI-assisted image interpretation. Concerns about the reliability of AI outputs and the potential for misinterpretation are recurrent themes in the literature. Blease et al. (2021) [18] emphasized the importance of transparent AI systems to build trust, suggesting that explainable AI could be crucial in ophthalmology as well.

### Qualitative insights and training needs

Qualitative responses in our study underscored the need for adequate training to mitigate the risk of misinterpreting AI outputs. This is supported by Meister et al. (2022) [9], who found that healthcare professionals often feel underprepared to integrate AI into their practice without proper education. The call for comprehensive training programs is a common recommendation in the literature, as seen in Meister et al. (2022) [9], who proposed structured curricula for AI literacy in medical education.

### Comparison with specific AI applications in glaucoma

Several studies have explored AI’s specific applications in glaucoma care, providing a benchmark for our findings. Hashemian et al. (2024) [19] demonstrated high accuracy in AI-based glaucoma detection using retinal images, yet clinician trust remained a hurdle. Asian cohorts report higher trust levels (30% vs. 7.5% here), possibly due to earlier AI exposure [17] [15], suggesting cultural factors at play. Our study’s emphasis on early screening aligns with Hashemian et al. (2024) [19], who highlighted AI’s potential in population-level glaucoma screening.

### Addressing barriers to AI integration

The identified barriers, including concerns about misinterpretation and insufficient training, are well-documented in the literature. Varkey et al. (2022) [12] [20] discussed the legal and ethical challenges of AI in healthcare, mirroring our respondents’ concerns about accountability. Meskó et al. (2022) [12] explored financial barriers to AI adoption, which were also mentioned in our qualitative responses.

### Regional context

Beyond replicating well-known demographic patterns, this study underscores the added value of examining AI adoption in the Bulgarian and broader Eastern European context. Compared to wealthier regions, limited healthcare expenditure, uneven access to OCT and perimetry outside academic centers, and the absence of national image repositories constrain readiness for AI implementation. Moreover, most existing glaucoma AI tools have been trained and validated on datasets from Western Europe or Asia, raising concerns about potential failure modes when applied in local practice. These region-specific barriers highlight the need for investment in digital infrastructure, development of locally representative datasets, and tailored implementation strategies to ensure safe and equitable AI adoption in Bulgarian ophthalmology.

This contextualization not only situates our findings within the global literature but also emphasizes the necessity of regional perspectives in shaping future policies and clinical strategies.

### Limitations

This study has several limitations that should be considered when interpreting the findings. First, the sample exhibited a predominance of early-career ophthalmologists and residents (36.5% with less than 5 years of experience; median age 35 years) and was female-dominant (73.1%). Such demographic skew may bias upward awareness and optimism regarding AI adoption, as younger clinicians are typically more familiar with digital technologies. However, exact population distributions of age, gender, and years of experience among Bulgarian ophthalmologists are not publicly available, which precluded post-stratification weighting or precise estimation of this potential bias.

Second, another limitation concerns the recruitment strategy. Although invitations were disseminated through professional societies and an online group with broad coverage of Bulgarian ophthalmologists and residents, participation was voluntary and the final sample was self-selected rather than randomly drawn. This introduces the possibility of selection bias, as individuals with greater interest in digital technologies or more active in professional networks may have been more likely to respond. Consequently, the achieved sample may not fully represent the demographic and attitudinal diversity of the national ophthalmologist population, and findings should be interpreted with this caveat in mind.

Third, the response rate of 31.2% raises concerns about non-response bias; although comparisons between early and late responders revealed no significant demographic differences, non-respondents might include individuals less interested in or more skeptical toward AI, potentially underrepresenting conservative viewpoints.

Fourth, the study’s generalizability is limited, as participants were primarily recruited through national and European ophthalmology networks, which may not fully capture perspectives from other regions or diverse socioeconomic contexts.

In addition, the cross-sectional design precludes assessment of longitudinal changes in perceptions, such as whether trust in AI evolves with greater exposure to clinical systems over time. Other issues include reliance on self-reported data, which is subject to social desirability bias (e.g., overstatement of positive expectations), the absence of measures for prior hands-on experience with AI in clinical practice (a potential confounder), and unmeasured urban–rural differences. Future studies should address these limitations by employing larger and more demographically balanced samples, longitudinal designs, and objective assessments of AI interaction to strengthen the robustness of conclusions.

### Future directions and recommendations

Future work should prioritize clinician-centered AI tools that are transparent, interpretable, and adapted to resource-limited healthcare systems. In Bulgaria and Eastern Europe, this requires cost-sensitive solutions, standardized electronic records, and the creation of locally representative image datasets to validate algorithms trained elsewhere. Pilot training initiatives, embedded in ophthalmology curricula and professional societies, could enhance clinicians’ confidence and readiness, while fostering interdisciplinary collaboration with technologists and ethicists. Strengthening digital infrastructure and addressing these regional constraints are essential steps toward safe and equitable AI adoption in glaucoma care.

### Addressing key concerns in AI integration

While our qualitative findings highlight concerns such as misinterpretation of AI outputs, insufficient training, and ethical/legal issues in glaucoma care, several strategies could mitigate these barriers. For misinterpretation, incorporating explainable AI (XAI) techniques, such as heatmaps for optic nerve analysis, enhances transparency. Advanced methodologies like Chain-of-Thought (CoT) prompting can break down AI reasoning step-by-step (e.g., evaluating intraocular pressure before optic damage assessment), making decisions more interpretable. Similarly, Retrieval-Augmented Generation (RAG) grounds AI in factual data from guidelines or datasets, reducing errors. In resource-limited Bulgarian settings, RAG could leverage local repositories for culturally relevant outputs [21].

To address insufficient training, structured curricula in ophthalmology programs should include hands-on AI simulations, with CoT and RAG integrated into educational tools for step-by-step glaucoma management training. Collaborations with the Bulgarian Ophthalmology Society could support workshops to build clinician confidence [22].

For ethical and legal issues, regular bias audits of AI models, using diverse datasets to ensure equity in Eastern European populations, are essential. Adherence to frameworks like the EU AI Act, with consent protocols and liability models, promotes accountability. CoT and RAG further enhance ethics by enabling traceable reasoning and reducing bias through diverse knowledge sourcing. Future pilots in Bulgaria could test these approaches to validate their impact on trust and adoption [22,23].

## Conclusion

Ophthalmologists see potential in AI for improving glaucoma management, particularly in diagnosis and screening, but current trust levels are low. Demographic factors—age, gender, and experience—shape these attitudes, highlighting the importance of addressing specific concerns to foster AI adoption. Future efforts should focus on developing reliable, clinician-friendly AI tools and providing comprehensive training to enhance trust and integration into glaucoma care.

## Supporting information

S1 Dataset Raw anonymized survey responses of Bulgarian ophthalmologists on AI and glaucoma care. (XLSX)

S1 Table Prevalence estimates of awareness, trust, expectations, and perceived applicability of AI, with 95% confidence intervals. (XLSX)

S1 Questionnaire The questionnaire, used in the study in English. (DOCX)
