# Supplementary material for: Awareness, trust, and expectations of AI for glaucoma care among Bulgarian ophthalmologists: Role of demographic factors
Source: PLOS Digit Health. 2026 Jan 22;5(1):e0001199. doi: 10.1371/journal.pdig.0001199 (PMC12826508; doi:10.1371/journal.pdig.0001199)
Supplement: S1 Questionnaire — (DOCX) [file pdig.0001199.s003.docx]

**Questionnaire AI and glaucoma in English**

**Demographics**

1. Biological sex

a) male

b) female

2. What is your specialty?

A) Eye disease specialist

B) Eye Disease Resident

C) Optician

D) optometrist

E) other

3. How many years of experience do you have?

A) < 5 years

B) 5-10 years

C) 10-20 years

D) > 20 years

**Glaucoma**

4. Do you know what glaucoma is?

A) Yes

B) No

5. Are you familiar with the symptoms and risk factors for the development of glaucoma?

A) Yes

B) No

6. How often do you meet patients with glaucoma?

A) Every day

B) Several times a week

C) Once a week

D) Once a month

E) Never

7. Do you treat patients with glaucoma?

A) Yes

B) No

**AI**

8. Do you know what "artificial intelligence" is?

A) Yes

B) No

9. Does artificial intelligence have a role in healthcare?A) Yes

B) No

C) I don't know.

10. Have you used artificial intelligence?

A) Yes

B) No

11. How often do you use artificial intelligence?

A) Every day

B) Several times a week

C) Once a week

D) Once a month

E) Never

12. Compared to your colleagues, how would you assess your knowledge of artificial intelligence and its application in the field of ophthalmology?

A) Excellent

B) Above average

C) At the intermediate level

(D) Below average

E) I'm not familiar with artificial intelligence

13. Do you doubt the diagnostic accuracy of artificial intelligence in the field of ophthalmology?

A) I don't trust artificial intelligence at all

B) I don't think I can fully trust AI

C) I don't fully trust AI

D) I have full confidence in AI

E) I cannot judge due to insufficient data

**AI and glaucoma**

14. Have you heard about the use of artificial intelligence for glaucoma screening and diagnosis?

A) Yes

B) No

15. Do you believe that artificial intelligence can improve the diagnosis and treatment of glaucoma?

A) I agree

B) I do not agree with

C) I don't know.

16. Do you trust the recommendations generated by artificial intelligence regarding the treatment of glaucoma?

A) Yes

B) No

C) I don't know.

**Future expectations**

17. After how long do you think artificial intelligence will have a significant impact in the field of ophthalmology?

A) It already has a significant impact on the ophthalmologist's practice

B) after 1 year

C) from 1 to 5 years

D) from 5 to 10 years

E) > 10 years

F) never

18. "I think AI will replace doctors."

A) I agree

B) Rather yes

C) I'm not sure

D) Rather not

E) I disagree

19. AI will change the doctor-patient relationship.

A) I agree

B) Rather yes

C) I'm not sure

D) Rather not

E) I disagree

20. The study of AI should also be included in medical education.

A) I agree

B) Rather yes

C) I'm not sure

D) Rather not

E) I disagree

21. AI will facilitate the practice of the modern ophthalmologist.

A) I agree

B) Rather yes

C) I'm not sure

D) Rather not

E) I disagree

22. What role do you think artificial intelligence should play in the future of ophthalmology (choose whatever you think is true)?

A) Screening of eye diseases

B) diagnosing eye diseases

C) tracking the progression of eye diseases

D) Detection of refractive errors

E) other

If you answered "other", please clarify.

____________________________________________________________________________________________________________________________________________________________________________________________________________________________________________________________________________________________________________

23. What do you think should be the main role(s) of artificial intelligence in the future of ophthalmology?

____________________________________________________________________________________________________________________________________________________________________________________________________________________________________________________________________________________________________________

24. How do you think artificial intelligence can improve the eyecare?

____________________________________________________________________________________________________________________________________________________________________________________________________________________________________________________________________________________________________________

25. What difficulties do you expect to face in the use of artificial intelligence in ophthalmology?

____________________________________________________________________________________________________________________________________________________________________________________________________________________________________________________________________________________________________________
